# Computer-Aided Design of Antimicrobial Peptides: Are We Generating Effective Drug Candidates?

**DOI:** 10.3389/fmicb.2019.03097

**Published:** 2020-01-22

**Authors:** Marlon H. Cardoso, Raquel Q. Orozco, Samilla B. Rezende, Gisele Rodrigues, Karen G. N. Oshiro, Elizabete S. Cândido, Octávio L. Franco

**Affiliations:** ^1^S-Inova Biotech, Programa de Pós-Graduação em Biotecnologia, Universidade Católica Dom Bosco, Campo Grande, Brazil; ^2^Centro de Análises Proteômicas e Bioquímicas, Pós-Graduação em Ciências Genômicas e Biotecnologia, Universidade Católica de Brasília, Brasília, Brazil; ^3^Instituto de Ciências Biológicas, Departamento de Biologia, Programa de Pós-Graduação em Ciências Biológicas (Imunologia/Genética e Biotecnologia), Universidade Federal de Juiz de Fora, Juiz de Fora, Brazil; ^4^Programa de Pós-Graduação em Patologia Molecular, Faculdade de Medicina, Universidade de Brasília, Brasília, Brazil

**Keywords:** computer-aided design, bacteria, biofilms, antimicrobial peptides, drug design

## Abstract

Antimicrobial peptides (AMPs), especially antibacterial peptides, have been widely investigated as potential alternatives to antibiotic-based therapies. Indeed, naturally occurring and synthetic AMPs have shown promising results against a series of clinically relevant bacteria. Even so, this class of antimicrobials has continuously failed clinical trials at some point, highlighting the importance of AMP optimization. In this context, the computer-aided design of AMPs has put together crucial information on chemical parameters and bioactivities in AMP sequences, thus providing modes of prediction to evaluate the antibacterial potential of a candidate sequence before synthesis. Quantitative structure-activity relationship (QSAR) computational models, for instance, have greatly contributed to AMP sequence optimization aimed at improved biological activities. In addition to machine-learning methods, the *de novo* design, linguistic model, pattern insertion methods, and genetic algorithms, have shown the potential to boost the automated design of AMPs. However, how successful have these approaches been in generating effective antibacterial drug candidates? Bearing this in mind, this review will focus on the main computational strategies that have generated AMPs with promising activities against pathogenic bacteria, as well as anti-infective potential in different animal models, including sepsis and cutaneous infections. Moreover, we will point out recent studies on the computer-aided design of antibiofilm peptides. As expected from automated design strategies, diverse candidate sequences with different structural arrangements have been generated and deposited in databases. We will, therefore, also discuss the structural diversity that has been engendered.

## Introduction

Peptides can be produced as part of the host defense system during infections (Hancock and Scott, 2000). Antimicrobial peptides (AMPs) belong to a diverse group of molecules produced by cellular tissues in a wide variety of organisms (Brogden, 2005). These peptides demonstrate potent antimicrobial activity and can readily be mobilized to neutralize a wide range of microbes, including viruses, bacteria, protozoa, and fungi (Shai, 2002). Moreover, this class of antimicrobials has shown promising endotoxin neutralization properties (Fleitas Martinez et al., 2019), which favors positive outcomes in animal models of sepsis. Finally, AMPs are known to have diverse modes of action depending on the bacterial targets they interact with and, therefore, are promising candidates for multi-target antibacterial treatments. Although these characteristics appear as promising features for drug development, some disadvantages have been pinpointed for AMP-based therapies, including chemical and physical instability (Zhao et al., 2016), proteolytic degradation (Pachon-Ibanez et al., 2017), short half-life and rapid elimination (Lombardi et al., 2015), slow tissue penetration (Koczulla et al., 2003), toxicity toward healthy human cells, and cell specificity (Oshiro et al., 2019). Based on that, an increasing number of computational strategies are underway, aiming at overcoming these obstacles by optimizing AMP sequences.

Advanced strategies of rational design allied to computational methods have been used for the development of more economical and powerful AMPs (Fjell et al., 2012). The rational design of new drugs has become a major area in medicinal chemistry, aiming at creating pharmaceutical products with greater specificity against microorganisms, along with reduced adverse effects (Porto et al., 2012). In this context, several computational tools have been developed to design AMP variants. Among them, we can mention empirical methods and machine learning, as well as stochastic approaches, which aim at the optimization of peptides through random processes (Porto et al., 2012). Machine learning models are useful for the efficient screening and optimization of a small number of sequences that could be further evaluated experimentally. Among the machine learning strategies, a particular focus has been given to the quantitative structure-activity relationship (QSAR) model (Mitchell, 2014), which uses physicochemical descriptors to predict the biological activity of peptides from their amino acid sequences (Hilpert et al., 2008).

In addition, *de novo* computational methods generate AMP sequences without a model sequence but using amino acid frequency and position preferences that can guarantee characteristics such as load, amphipathicity, and structure (Porto et al., 2012). This method has allowed the generation of multiple sequences with a great diversity of amino acid composition, tridimensional structures, and mechanisms of action (A Hiss et al., 2010). Based on the *de novo* model, an increasing number of tools have been developed, including linguistic models. According to Loose et al. (2006), AMPs can be designed through a formal language, consisting of vocabulary (e.g., amino acid residues) and rules (e.g., amino acid patterns). Therefore, by using this “grammar” model, it is proposed that AMPs could act more specifically by recognizing intracellular targets or acting directly on bacterial membranes. More recently, this model was further explored by associating the identification of amino acid patterns in public databases, followed by their insertion into a peptide sequence (AMP or not) aiming at generating optimized AMPs (Porto et al., 2018a).

Apart from the computational methodologies cited above, genetic algorithms appear as an alternative in the development of new drugs. Evolutionary methods rely on genetic algorithms to produce successive generations of mutations and deletions in a target sequence to improve fitness and identify determinants that confer antibacterial activity, for instance, through activity prediction methods (Kliger, 2010; Fjell et al., 2011). Over generations, the sequences are evaluated and those with lower fitness values are removed from the candidate sequences, thus generating more specific candidates for the desired function (Fjell et al., 2011).

**FIGURE 1:**
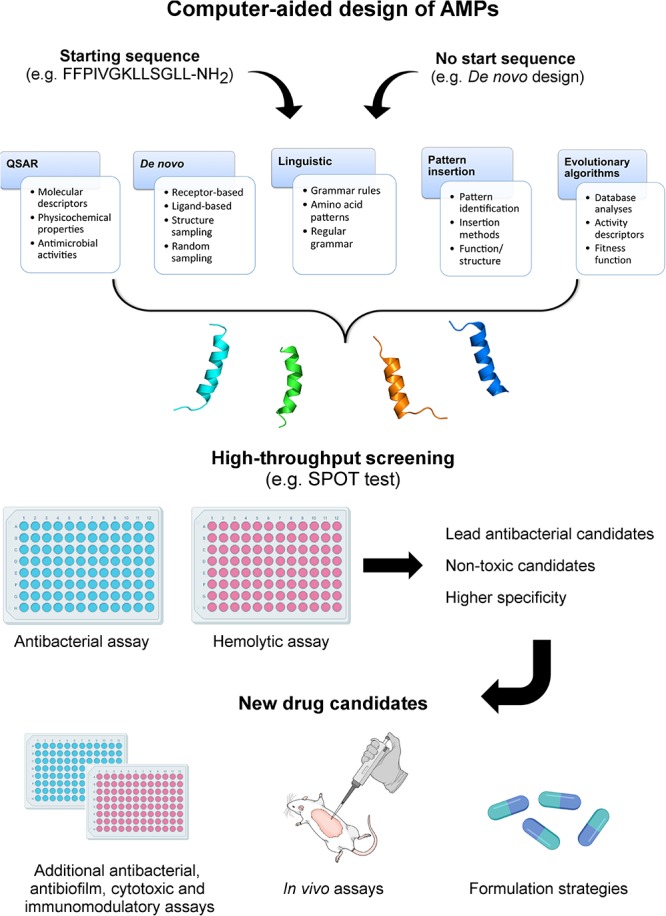
Computer-aided design of AMPs. In this review, five different methods for computationally designing AMPs are described, including QSAR, *de novo*, linguistic, pattern insertion, and evolutionary/genetic algorithms. The computer-aided design of AMPs may start from *de novo* methods (no seed sequence) or based on known peptides aiming at generating optimized analogs. Depending on the strategy, different parameters will guide the design, including molecular and activity descriptors, tridimensional structures, grammar rules, pattern identification (motifs), and fitness functions. From this point, diverse candidate sequences are generated and further submitted to structure prediction and screening for antibacterial and hemolytic properties. The lead candidates are then submitted to in-depth functional and structural analyses, including antibacterial, antibiofilm, immunomodulatory, and *in vivo* assays. Ultimately, different AMP formulation strategies are investigated, aiming at optimizing the evaluation of these peptide-based drugs in advanced clinical trials.

Although different computational methods have been used to predict and generate optimized AMP sequences (Table 1), a crucial question remains: are we generating effective drug candidates? Here we have focused on the primary computational methodologies applied for computationally designing AMPs (Figure 1) and analyze how effective these new drug candidates have been against bacteria, biofilms and animal infection models. We also describe the structural diversity that has been generated by the automated design of AMPs and how this feature influences the antimicrobial properties of these molecules.

**TABLE 1:** Summary of AMP databases and computational tools for designing and predicting AMP sequences.

**AMP databases**	**Description**	**Link**
Antimicrobial Peptide Database (APD)	Comprehensive database for AMPs, including searching tools, calculation and prediction, peptide design, 3D structures, and classification	http://aps.unmc.edu/AP/main.php
Collection of Anti-Microbial Peptides (CAMP_R__3_)	Created to expand and accelerate antimicrobial peptide family-based studies. Includes AMPs prediction tools (SVM, ANN, DA, and RF), sequence alignment, pattern creation, and HMMs-based search	http://www.camp.bicnirrh.res.in
Yet Another Database of Antimicrobial Peptides (YADAMP)	The main difference between YADAMP and other web databases of AMPs is the explicit presence of antimicrobial activity against the most common bacterial strains. Includes segment search, structure information, peptide mapping, and sequence similarity	http://www.yadamp.unisa.it
Biofilm-active AMPs database (BaAMPs)	First database dedicated to AMPs specifically tested against microbial biofilms. Includes peptide list, experiment list, sequence alignment, and physicochemical descriptors calculator	http://www.baamps.it
**AMP prediction and design**		
*i*AMPpred	SVM for predicting AMPs and non-AMPs. Three different categories of features has been used, including compositional, structural, and physicochemical features	http://cabgrid.res.in:8080/amppred/
iAMP-L2	A two-level multi-label classifier for identifying antimicrobial peptides and their functional types	http://www.jci-bioinfo.cn/iAMP-2L
AMPep	Sequence-based prediction of antimicrobial peptides using distribution patterns of amino acid properties and RF	https://omictools.com/ampep-tool
Mutator	A computational tool for predicting how single or double amino acid substitutions could improve the therapeutic index of helical AMPs	http://split4.pmfst.hr/mutator/
AntiBP2	Predicts the antibacterial peptides in a protein sequence. Prediction can be done by using SVM-based method using coposition of peptide sequences and overall accuracy of this server is ∼92.14%	http://crdd.osdd.net/raghava/antibp2/
ClassAMP	Uses RF and SVM to predict the propensity of a protein sequence to have antibacterial, antifungal, or antiviral activity	http://www.bicnirrh.res.in/classamp/
AMPA	Web tool for assessing the antimicrobial domains of proteins, with a focus on the design on new antimicrobial drugs	http://tcoffee.crg.cat/apps/ampa/do
DBAASP	Provides users with information on detailed structure (chemical, 3D) and activity for those peptides, for which antimicrobial activity against particular target species have been evaluated experimentally	https://dbaasp.org/home
Joker	An algorithm to design antimicrobial peptides using their language	https://github.com/williamfp7/Joker

*AMP: antimicrobial peptide; SVM: support vector machine; RF: random forest; DA: discriminant analysis; ANN: artificial neural network; HMM: hidden Markov models.*

## Computational Methods for Designing Amps

### Machine Learning (With a Particular Focus on QSAR)

Machine learning is considered a smart and efficient method for computer-made decisions based on unseen data, learning from extensive and comprehensive training data (Jia et al., 2015). In this context, different algorithms have been developed based on machine learning methods, including support vector machine (SVM), fuzzy K-nearest neighbor (FKNN), random forest (RF), and neural network (NN) (LeCun et al., 2015).

Support vector machine is an algorithm for maximizing a particular mathematical function concerning a given collection of data (Noble, 2006). This algorithm has been used as a prediction tool that considers peptide amino acid composition, physicochemical properties, and structural features as parameters to classify AMPs with high accuracy (e.g., iAMPpred – Table 1) (Meher et al., 2017). Moreover, SVM has also been used to map membrane activity in undiscovered peptide sequences (Lee et al., 2016). When it comes to pattern recognition, the K-nearest neighbor (KNN) method has been considered the most straightforward algorithm, from which the FKNN method is derived (Sim et al., 2005). Xiao et al. (2013) have developed a two-level multi-label classifier, named iAMP-2L, to predict AMPs and their activities (Table 1). An improved FKNN method was applied for AMP classification, followed by regular multi-label learning processing (Xiao et al., 2013). As a result, this method not only allowed the identification of potential AMP sequences but also classified these sequences according to five different function types (Xiao et al., 2013). AMP prediction has also been performed by RF methods, which are based on ensemble learning algorithms and work by multiple decision trees built on training data (Schierz, 2009).

In terms of AMP prediction, studies have proposed a new tool called AmPEP (Table 1), as an attempt to develop a highly accurate RF classifier for AMP prediction based on pattern distribution and physicochemical properties (Bhadra et al., 2018). Its performance was comparable with other predictive tools and it showed higher values for particular parameters of comparison, even with a reduced number of features. Finally, NN comprises estimators of universal function that have been used to identify patterns into sequences, and also to build structure-function relationships. This model consists of many simple, connected processors called neurons, each producing a sequence of real-valued activations (Schmidhuber, 2015). In AMP design, NN has been applied in evolutionary and genetic algorithms (Schneider et al., 1998; Fjell et al., 2011), as well as random sequence generation (Cherkasov et al., 2009) of AMP candidates based on an initial model sequence (aiming at optimization for a particular function) or *de novo* (no template sequence).

Apart from the approaches cited above, QSAR models have been pinpointed as highly effective in predicting models based on biological behavior (Lo et al., 2018). QSAR models were developed to discover efficient and robust computational procedures to locate molecules with known activities or properties in databases and virtual libraries (Golbraikh et al., 2017). A QSAR model is a simple mathematical relationship derived from a set of training molecules with known properties using regression or classification-based approaches (Roy et al., 2015). This technique offers an *in silico* tool for the development of predictive models toward various activity and property endpoints of a series of chemicals using response data and molecular structure information (Walker et al., 2003). In this context, QSAR models can be used to identify determinants that are important for antimicrobial activities, and then use these determinants to design new, more effective AMPs (Lee et al., 2017). Moreover, although most QSAR methods are focused on antibacterial activities, some works have considered the antimicrobial potential of a drug candidate and its toxicity, simultaneously, to obtain improved pharmacological profiles (Cruz-Monteagudo et al., 2011). This approach has been applied, for instance, to generating thrombin and trypsin inhibitors, including fluoroquinolones (Cruz-Monteagudo et al., 2008), 3-amidinophenylalanine inhibitors of Nicolotti et al. (2009), and AMPs (Cruz-Monteagudo et al., 2011).

For instance, QSAR models have been used to calculate the antibacterial activity of mastoparan analogs derived from wasp venom, based on descriptors derived from the simple representations of peptides as a sequence of amino acids (Table 2) (Toropova et al., 2015). More recently, Czyzewski et al. (2016) reported a model based on the QSAR algorithm, which appeared to predict peptoid (AMP mimics) antibacterial activity accurately, based on the analysis of a set of structurally diverse peptoids (Table 2) (Czyzewski et al., 2016). An increase in AMP selective index has also been achieved by QSAR methods, including a computational tool called Mutator (Table 1). It suggests residue variations by improving peptide selectivity through appropriate mutations, limited to one or two amino-acid substitutions based on QSAR criteria (Table 2) (Rončević et al., 2019). Interestingly, QSAR has also been used to identify motifs in coiled-coil peptides aiming at facilitating the production of silver nanoparticles forming peptides with antibacterial potential (Table 2) (Bozic Abram et al., 2016). Anti-tuberculosis (anti-TB) AMPs have been developed using QSAR methods. For instance, Nurbo et al. (2007) described the synthesis of *Mycobacterium tuberculosis* ribonucleotide reductase (RNR) peptide inhibitors. These peptides were initially submitted to an alanine scan and, based on their results, it was found that Trp5 and phe7 are crucial residues for anti-TB properties. Moreover, a QSAR model was developed based on the heptapeptides synthesized, revealing the positive influence of negatively charged residues at positions 2, 3, and 6 on the peptides’ inhibitory potential toward *M. tuberculosis* (Nurbo et al., 2007). Finally, as described above, the multiobjective optimization of AMPs has gained attention over the years and can be used, for instance, as an approach for jointly handling potency and toxicity in computer-made AMPs. Therefore, Cruz-Monteagudo et al. (2011) developed a multicriteria QSAR model for handling the antibacterial and hemolytic activities of cyclic β-hairpin cationic peptidomimetics (Cβ-H). Along with this multicriteria method, which presented ∼80% accuracy in training and external validation sets, virtual screenings for identifying selective antibacterial Cβ-HCPs were carried out (Cruz-Monteagudo et al., 2011). Thus, this study reveals the advantages of multicriteria methods as promising chemoinformatics to generate selective AMPs with a higher therapeutic index.

**TABLE 2:** Summary of the computer-aided designed AMPs here described in terms of antibacterial potential, target bacterial species, and structural profile.

**Computer-aided design method**	**Peptide name**	**Treatment strategy**	**Target bacteria**	**Structural profile**	**References**
QSAR	Mastoparan-analogs (MP to MP6; PDDA to PDDA-12; PDDB to PDDB-5; PMM to PMM-14); peptoid 1; dadapin-1 to -8; P4C2; IDR-3002	Bacteriostatic; bactericide; antibiofilm; anti-infective (murine invasive *S. aureus*); induction of silver nanoparticles formation for combating planktonic bacteria	*A. baumannii*; *E. cloacae*; *E. coli*; *K. pneumoniae*; MRSA; *M. tuberculosis P. aeruginosa*; *P. maltophilia*; *S. aureus*	α-helix; β-hairpin-like; random coil	Nurbo et al., 2007; Toropova et al., 2015; Bozic Abram et al., 2016; Czyzewski et al., 2016; Haney et al., 2018; Rončević et al., 2019
*De novo*	Peptide 1 to 5; LDKA; DFTamP1; SP1 to SP15; SPD1 and SPD15	Bacteriostatic	*Acinetobacter* sp.; *B. subtilis*; *E. aerogenes*; *E. coli*; *E. faecalis*; *E. faecium*; *K. pneumoniae*; *P. aeruginosa*; *S. aureus*; *S. cohnii*; *S. epidermidis*; *S. haemolyticus*; *S. hominis*; *S. warnerii*	α-helix; random coil	Mishra and Wang, 2012; Faccone et al., 2014; Chen et al., 2019; Vishnepolsky et al., 2019
Linguistic model	D28, R8 and D51; NN2_0018 and NN1_0050	Bacteriostatic; bactericide; anti-infective (mouse peritoneal model of infection with carbapenem-resistant *A. baumannii*)	*A. baumannii*; *B. alcalophilis*; *B. anthracis*; *B. bronchiseptica*; *B. cereus*; *C. freundii*; *C. glutamicum*; *C. koserii*; *C. pseudoTB*; *E. aerogenes*; *E. coli*; *E. faecalis*; *E. gergoviae*; *H. influenza*; *K. pneumoniae*; *K. oxytoca*; *M. luteus*; MRSA; *N. mucosa*; *P. aeruginosa*; *P. mirabilis*; *P. vulgaris*; *S. aureus*; *S. enterica*; *S. boydii*; *S. flexnerii*; *S. haemolyticus*; *S. maltophilia*; *S. typhimurium*; *V. cholera*	α-helix; random coil	Loose et al., 2006; Nagarajan et al., 2018
Pattern insertion algorithm	EcDBS1R6; PaDBS1R6 and PaDBS1R1; PaDBS1R6F10; mastoparan-R1 and R4	Bacteriostatic; bactericide; antibiofilm; skin infection treatment (skin scarification mouse model); anti-bacteremia	*A. baumannii*; *E. cloacae*; *E. coli*; *E. faecalis*; *K. pneumoniae*; *MRSA*; *P. aeruginosa*; *S. aureus*	α-helix; random coil; β-turn	Cardoso et al., 2018a; Cândido et al., 2019; Fensterseifer et al., 2019; Oshiro et al., 2019
Evolutionary algorithm	GN-1 to GN14; Guavanin 1 to 12; GMG_01, GMG_02, GMG_01_SCR, GMG_03, CM18, CM12 and GMG_05Z; temporin-Ali analogs	Bacteriostatic; bactericide; skin infection treatment (skin scarification mouse model)	*A. baumanni*i; *E. coli*; *E. faecium*; *K. pneumoniae*; *P. aeruginosa*; *S. aureus*; *S. pyogenes*	α-helix; random coil	Fjell et al., 2011; Maccari et al., 2013; Porto et al., 2018b; Yoshida et al., 2018

It is estimated that bacterial biofilms account for ∼80% of microbial infections in humans (Magana et al., 2018). Nevertheless, although many efforts have been made in the past decade to counter biofilm infections, we still lack effective commercial drugs that were designed to treat biofilms (most treatments include the use of conventional antibiotics designed to target planktonic bacteria). In the field of designed AMPs, some candidates have shown promising antibiofilm properties. However, the mechanisms by which AMPs inhibit biofilm formation or eradicate pre-formed biofilms are still under investigation. Moreover, as for conventional antibiotics, antibiofilm AMPs are usually designed to target planktonic bacteria and, sometimes, also present antibiofilm potential. Therefore, we still lack knowledge on the determinants that rule AMP antibiofilm activities.

In this context, Haney et al. (2018) performed the computer-assisted (QSAR) discovery of peptides that specifically act on bacterial biofilms. In that work, a peptide library was built based on the immunomodulatory and antibiofilm peptide, IDR-1018 (Haney et al., 2018). A total of 96 single amino-acid-substituted variants of IDR-1018 were submitted to high-throughput screening for antibiofilm activities against methicillin-resistant *Staphylococcus aureus* (MRSA) biofilms. Based on the *in vitro* results, QSAR models were used to correlate the antibiofilm potential of these variants with the descriptors derived from their sequences. Novel variants were generated using a 3D QSAR model to predict the probability of a peptide to present antibiofilm activity from a virtual library of 100,000 peptides. A sub-set of these peptides were then synthesized and their antibiofilm properties evaluated, resulting in ∼85% prediction accuracy. Among all peptides generated, IDR-3002 was eightfold more potent against resistant bacterial biofilm than the parent peptide IDR-1018, thus demonstrating the potential of using this strategy to design biofilm-active peptides (Table 2) (Haney et al., 2018). Although this study introduces a promising strategy for the computer-aided design of improved antibiofilm peptides, it is worth noting that the modeling strategy used only classifies peptide candidates as “active” or “inactive,” but it does not consider antibiofilm potency. In addition, the extension of this selective antibiofilm property should also be investigated against other bacterial strains to clarify whether the designed peptides are strain selective or not. Thus, as a strategy to overcome these obstacles, the authors anticipate that it is possible to iteratively improve the QSAR models for antibiofilm peptides, as an increasing number of sequences have been deposited in databases, which in turn allows more accurate predictions (e.g., BaAMPs: the database of biofilm-active AMPs – Table 1).

### *De novo* Computational Design

The concept of computer-aided *de novo* drug design was first introduced more than 25 years ago (Danziger and Dean, 1989). In that study, an algorithm for knowledge acquisition about hydrogen-bonding regions on protein surfaces was generated, aiming at designing novel ligands that specifically bind to a target site. Ever since, diverse *de novo* algorithms have been reported and many feasible drug candidates have been generated, and vast libraries (from 10^4^ to 10^6^ compounds) are usually screened in biological assays (Dobson, 2004; Schneider and Fechner, 2005). In general, *de novo* drug design methods are based on the candidate drug assembly, its quality in terms of the desired function, and, finally, the search space sampling based on the given information (e.g., physicochemical principles, descriptors, and chemical structure) (Schneider and Fechner, 2005).

Among the inputs required for *de novo* drug design, the primary target constraints are of high relevance, as they determine the structural-guided generation of novel candidates. Therefore, receptor-based and ligand-based *de novo* designs have often been used when structural information is available. It allows the prediction of interaction sites between the target molecule and the designed drugs, as well as providing receptor-based and ligand-based scorings to select the best candidates (Figure 1) (Miranker and Karplus, 1995; Pearlman and Murcko, 1996; Pierce et al., 2004; Schneider and Fechner, 2005). In addition, secondary target constraints consist of those other than binding affinity. They include structure sampling, random sampling, combinatorial search strategies (e.g., heuristic algorithms), and evolutionary algorithms (Douguet et al., 2000; Schneider and Fechner, 2005).

The computational *de novo* design of AMPs is usually experimentally characterized only if structure-prediction calculations that start from the designed sequence strongly converge on the designed structure (Huang et al., 2016). Therefore, AMP *de novo* design is a possible way to explore the number of new sequences and small subsets. Furthermore, the exploration of AMP *de novo* design with synthetic biology concepts represents a promising scenario for the development of foldamers and biomimetic antimicrobial polymers that mimic AMPs for therapeutic purposes (Tew et al., 2002; Choi et al., 2009; Woolfson et al., 2015).

Different strategies using *de novo* algorithms have been demonstrated by Faccone et al. (2014), aiming at generating effective drug candidates (Faccone et al., 2014). Those authors have engineered AMPs based on the omiganan (MBI-226) peptide using a combination of computer-assisted approaches. They settled specific amino acid positions and identified functionally relevant motifs in natural or designed peptides. By applying these parameters, five cationic α-helical peptides were designed, synthesized, and tested against *Pseudomonas aeruginosa*, *Escherichia coli*, *S. aureus*, and three different strains of *Enterococcus faecalis*, alongside 39 Gram-positive and 43 Gram-negative isolates with different resistance mechanisms. It was observed that *de novo* designed peptides 1, 2, and 5 showed similar or enhanced antimicrobial activity compared with omiganan against five of the tested strains. The authors concluded that peptides 1, 2, and 5 showed the best antibacterial activity against a broad spectrum of clinical isolates, thus encouraging their use as template molecules for new drugs (Table 2).

Database filtering technology (DFT) has also been proposed as a promising approach to retrieve the most probable parameters from the AMP Database (APD – Table 1) (Wang et al., 2016) for the *de novo* design of improved AMPs. Mishra and Wang (2012) first introduced this concept. In that work, the authors used peptide activity, peptide length, amino acid frequency, charge, hydrophobicity, structure profile, and motifs as filters for designing a novel peptide, named DFTamP1. This peptide effectively inhibited an MRSA strain (MIC = 3.1 mM). Moreover, MRSA cells treated with DFTamP1 at 2 × MIC (6.2 μM) were completely killed after 60 min. The mechanism by which DFTamP1 kills MRSA was also elucidated, revealing a surface-associated mechanism that leads to cell leakage (Mishra and Wang, 2012). DFTamP1 has high similarity with temporins from amphibians, which usually present a proline residue at the N-terminal region. Therefore, by replacing the Ser4 in DFTamP1 by a proline, the authors also observed a gain of function toward *Bacillus subtilis* and *E. coli*. Taken together, all these findings reveal the importance of this database-derived molecular design concept (DFT) as a suitable strategy for generating peptide-based antibiotics. For a more extensive review on database-guided discovery and design of therapeutic peptides, please see Wang (2013).

More recently, Chen et al. (2019) reported the molecular dynamics (MD)-guided *de novo* design of a 14-amino acid residues peptide, which is constituted of only four amino acid types (LDKA), and derived from a polyleucine peptide (GL_5_KL_6_G) (Chen et al., 2019). The LDKA peptide was tested against *E. coli*, *S. aureus*, and *P. aeruginosa*, revealing MIC values from 10 to 66 μM (Table 2). This antibacterial efficacy is directly correlated with the membrane pore formation mechanism displayed by LDKA, which forms large pores at a low peptide-to-lipid ratio, thus opening a new door for the optimization of short, pore-forming AMPs.

The development of a novel AMP prediction tool, called Special Prediction (SP), has recently allowed the generation of a new algorithm (DSP) to design AMPs through *de novo* methods. DSP has been used to computationally design short AMP candidates with high therapeutic indexes and promising effects on Gram-negative bacteria. Based on that, Vishnepolsky et al. (2019) reported that, among the 15 DSP designed AMPs, 14 had their antibacterial efficacy confirmed experimentally against *E. coli*. In addition, these peptides demonstrated high antimicrobial activity against *P. aeruginosa* and *Acinetobacter baumannii* pathogens (Vishnepolsky et al., 2019). Considering the obstacle imposed by AMP degradation when administrated in animal models, D-enantiomer analogs were also generated in that study and one synthetic D-peptide (SP15D) revealed the highest antibacterial effects toward *E. coli*, with MIC values ranging from 0.2 to 0.9 μM (0.39 to 1.56 μg.mL^–1^). In addition, as expected for D-peptides, SP15D revealed improved resistance to proteolytic degradation, along with a mechanism of action similar to those reported for cell-penetrating peptides. It was also highlighted that SP15D constitutes a select group of highly active (lowest MIC value) short AMPs deposited in the Database of Antimicrobial Activity and Structure of Peptides (DBAASP), rendering this AMP a promising candidate for more advanced trials (Table 2).

### Linguistic Model

The linguistic model has attracted attention in the last decade, as it considers amino acid sequences as a formal language that could be described by a set of regular grammars (Loose et al., 2006). Therefore, the linguistic model opens a new perspective concerning the physicochemical-guided design of AMPs, as it proposes that each amino acid represents a “word” that should be placed in the right position for the “phrase” (sequence) to make sense. The grammar rules that govern, for instance, the amphipathic character of most AMPs are usually the repeated usage of amino acid sequences (patterns), which are commonly found in many naturally occurring AMPs, including cecropin from insects (Van Hofsten et al., 1985) and brevinin from amphibians (Simmaco et al., 1994). Thus, considering the increasing number of AMP sequences deposited in public databases, along with the availability of pattern recognition computational tools, it is expected that AMP patterns (or, in other words, set of regular grammars) could be retrieved from large data sets and further incorporated in template sequences to design improved AMPs (Figure 1).

The above-cited principles were first described by Loose et al. (2006). In that work, the authors retrieved a set of 684 regular grammars (patterns) from 526 well-known AMPs deposited in APD (Table 1) (Wang et al., 2016). From this point on, the overlapping grammar rules were put together, thus generating 20-amino-acid residue sequences incorporating the antimicrobial syntax. After clustering, a total of 42 sequences were selected for antibacterial assays (Loose et al., 2006). Moreover, shuffled sequences were also synthesized, comprising peptides with the same amino acid composition as their parent peptides, but arranged randomly and thus being “ungrammatical.” It was hypothesized that, despite conserving the physicochemical characteristics of their parent peptides, the shuffled variants would not have antibacterial properties. Among the peptides generated by this method, 18 were capable of inhibiting *E. coli* and *Bacillus cereus* growth. The two most promising candidates, D28 and D51, inhibited *B. cereus* at 16 μg.mL^–1^. Only two out of the 42 shuffled sequences presented antibacterial activity. D28, the best candidate, was also shown to inhibit *S. aureus* and *Bacillus anthracis* at 16 μg.mL^–1^ (Table 2) (Loose et al., 2006).

Considering the highest antibacterial activity of D28, this peptide was submitted to a heuristic approach by inserting mutations aiming at the modulation of physicochemical properties (e.g., charge, hydrophobicity, and hydrophobic moment) and removal of proline residues from the final candidate sequences. A total of 44 candidates were generated, among which the D28 variants, including an internal proline mutation by lysine or glycine, presented the highest activities toward *S. aureus*, *E. coli*, and *B. cereus* (Table 2) (Loose et al., 2006). In conclusion, this method for designing novel AMPs appears as a suitable methodology that does not require structure-function data or time-consuming structural-based approaches through complex peptide/target simulations.

Similarly, Nagarajan et al. (2018) reported the computational design of novel AMPs through a long short-term memory (LSTM) language model (Nagarajan et al., 2018). In that work, the YADAMP (yet another database of AMPs) database (Table 1) was used to retrieve AMP patterns and train the LSTM model. A total of 30,832 peptides were generated by the LSTM model, among which 17,390 remained after removing redundant and >30 residues sequences. Moreover, after filtering for cationic, amphipathic peptides, 6415 sequences were obtained, from which the 10 best (lowest predicted MIC) were selected for chemical synthesis (Nagarajan et al., 2018). These peptides were initially evaluated against *E. coli*, among which four presented MIC values < 10 μM. The most effective peptides, named NN2_0018 and NN1_0050, were active against a series of multidrug-resistant clinical isolates from 4 to 128 μg.mL^–1^, including *E. coli*, *A. baumannii*, *Klebsiella pneumoniae*, *P. aeruginosa*, and *S. aureus* (Table 2). These peptides also inhibited the growth of MRSA and carbapenem-resistant strains. When evaluated *in vivo* using a mouse peritoneal model of infection with carbapenem-resistant *A. baumannii*, the peptide NN2_0018 was proved to significantly reduce the bacterial load (100 times) compared with mice treated with meropenem. Finally, both NN2_0018 and NN2_0050 interacted and disrupted bacterial membranes. Moreover, NN2_0018 also caused secondary systemic effects on bacteria, as this peptide interfered with gene-regulation (Nagarajan et al., 2018). Taken together, these findings revealed the effectiveness of applying the linguistic model in AMP design, also highlighting the importance of combining computational strategies to achieve more effective drug candidates.

### Automated Amino Acid Patterns Inserted Into Sequences

The tridimensional structure of peptides/proteins provides useful information about the molecular basis of their biological function (Chen and Bahar, 2004). Therefore, peptide/protein functions are associated with a particular sequence or structural motifs, and the identification of functional patterns and their role (Chen and Bahar, 2004). In this context, once a functional or structural pattern is identified from a model sequence, it can be inserted into a target sequence, aiming at generating novel biological functions (Figure 1). Among the insertion methods, the sliding window considers the aggregation propensity of amino acid sequence segments of various lengths (Trainor et al., 2017).

Based on these principles and considering the linguistic models described above, Porto et al. (2018a) hypothesized that, if an AMP is constituted of a combination of patterns, then the addition of an antimicrobial pattern to a peptide sequence (AMP or not) would generate or improve AMPs (Porto et al., 2018a). Based on that, a novel rational design algorithm was developed, named Joker (Table 1). This algorithm performs modifications on peptide sequences based on the insertion of antimicrobial patterns in a non-cumulative way, using a sliding window system (Porto et al., 2018a). Regarding Joker’s accuracy, the authors observed that among 84 designed AMPs, 55 were active against bacteria, representing a rate of 65% of accuracy.

Recently, Joker was used to design nine variants through the insertion of the α-helical pattern (KK[ILV]x_(__3__)_[AILV]) into a fragment from the mercury transport protein MerP (MKKLFAALALAAVVAPVW) from *E. coli* (Porto et al., 2018a). This pattern was retrieved from 248 α-helical AMPs deposited in the APD (Wang et al., 2016). Among the variants generated, the fifth peptide sequence, named EcDBS1R5 (*E. coli* database sequence – EcDBS), was studied by Cardoso et al. (2018a). This peptide showed potent antibacterial activity against susceptible and resistant bacterial strains, with MIC values from 2–16 μM for Gram-negative strains and from 8–32 μM for Gram-positive strains (Table 2) (Cardoso et al., 2018a). This peptide also displayed antibiofilm properties, as EcDBS1R5 was capable of dispersing two-day-old *P. aeruginosa* biofilms (at a concentration of 16 μM), also reducing the viability of biofilm cells, but not completely eradicating the preformed biofilm. In addition, this peptide showed no cytotoxicity toward non-cancerous and cancerous cell lines. Nevertheless, EcDBS1R5 displayed anti-infective activity *in vivo*, decreasing *P. aeruginosa* colony counts by two-logs at 2 days post-infection in a scarification skin infection mouse model (Table 2) (Cardoso et al., 2018a).

Another template sequence identified by Joker corresponds to a ribosomal fragment (MARNKPLGKKLRLAAAFK) from the archaeon *Pyrobaculum aerophilum*. From this sequence (by inserting the α-helical pattern described above), the variants PaDBS1R1 and PaDBS1R6 were generated. PaDBS1R1 displayed potent antibacterial activity, with low micromolar MIC values ranging from 1.5 to 12.5 μM against Gram-negative and Gram-positive bacteria (Table 2) (Irazazabal et al., 2019). Moreover, the PaDBS1R6 peptide, which was also tested against Gram-negative and Gram-positive bacteria of clinical interest, proved to be selective for Gram-negative strains (Table 2). PaDBS1R6 was also active against *P. aeruginosa* preformed biofilms, reducing its volume at 16 μM. The *in vivo* effectiveness of these peptides was further evaluated using a scarification skin infection mouse model. A single dose (64 μM) of PaDBS1R6 was capable of reducing the initial bacterial load (∼10^8^ CFU.mL^–1^) by up to three orders of magnitude after 2 days of treatment (Table 2). However, after four days of infection, the bacterial load increased for both peptide-treated and control animals, which might be related to degradation events *in vivo* (Fensterseifer et al., 2019).

Based on the antimicrobial potential exhibited by the peptide PaDBS1R6 (Fensterseifer et al., 2019), and aiming at reducing this peptide’s size, Cândido et al. (2019) performed sliding window analysis, thus generating ten fragments derived from PaDBS1R6. As a result, the sliding window fragment PaDBS1R6F10 was the most active peptide at inhibiting bacterial growth, displaying activity toward *E. coli* (16–32 μM) and *E. faecalis* (4–8 μM) strains (Table 2). In contrast, *S. aureus* and *P. aeruginosa* were inhibited only at the highest concentration tested (32 μM). PaDBS1R6F10 was also proved to kill *P. aeruginosa* biofilm-constituting cells at 16 μM. Nonetheless, this peptide is not capable of completely eradicating *P. aeruginosa* biofilms. This peptide, which showed no cytotoxic activity against mammalian cells, was tested *in vivo* in the same mouse model described above (skin infection). PaDBS1R6F10 decreased the bacterial load gradually, reaching a reduction of ∼10^3^ CFU.mL^–1^ after 4 days of treatment. Interestingly, this derivate exhibit improved *in vivo* activity when compared to the parental peptide PaDBS1R6, which did not maintain its anti-infective efficacy *in vivo* on the fourth day (Fensterseifer et al., 2019). According to the authors, this might suggest that the *in vivo* activity of these peptides (PaDBS1R6 and PaDBS1R6F10) is time-dependent and possibly involves peptide degradation events. Therefore, it is possible that the PaDBS1R6R10 peptide, as a short fragment (10-amino acid residues), has a higher resistance to enzymatic degradation *in vivo* (fewer cleavage sites) when compared to the 19-amino acid residues parental peptide PaDBS1R6.

Cytotoxicity remains a fundamental feature in peptide design (Torres et al., 2019). Bearing this in mind, Oshiro et al. (2019) recently used a peptide sequence (mastoparan-L) isolated from the wasp venom *Vespula lewisii* (Hirai et al., 1979) as input for the Joker algorithm aimed at reducing the hemolytic and cytotoxic effects of this peptide, as well as improving/extending its antibacterial properties (Table 2). In that work, five analog sequences were obtained by inserting the α-helical pattern (KK[ILV][AL]x[RKD][ILV]xxKI). Among them, the variants R1 and R4 showed improved antibacterial activities and cell selectivity when compared to the parental peptide (mastoparan-L). R1 and R4 were capable of inhibiting the growth of Gram-negative and Gram-positive bacterial strains with MICs ranging from 2 to 8 μM; however, contrary to the parent peptide, these variants were non-toxic on mammalian cells. In addition, R1 and R4 were capable of eradicating *P. aeruginosa* preformed biofilm at 16 μM. These two variants also demonstrated *in vivo* anti-infective activity in a *P. aeruginosa* skin infection mouse model. After a single dose of 64 μM, both the parent peptide and variants reduced the initial bacterial burden (∼100-fold reduction) 2 days post-infection (Table 2). However, on day 4, the effectiveness of the parental peptide and R4 decreased, whereas the variant R1 reduced the bacterial count 1000 times.

### Evolutionary/Genetic Algorithms

Evolutionary/genetic algorithms constitute an approach that has been used to classify virtually any new AMP sequences through fitness functions based on activity descriptors and information collected from APDs (Figure 1) (Torres and De La Fuente-Nunez, 2019). In AMP design, the simultaneous optimization of two or more characteristics may be required (e.g., sequence length or a particular amino acid composition) and, therefore, multiobjective evolutional algorithms can be employed to provide an optimal solution (Maccari et al., 2015). Therefore, AMP design through this method results from molecular evolution, which in part is driven by random and parsimonious changes of amino acid sequences and by subsequent natural selection for the stringent functionality of folded AMP molecules (Motomura et al., 2012). Moreover, this method is based on optimization processes combined with machine learning methods to provide more efficient antimicrobial predictions when the next generation of candidate sequences is analyzed. Thus, despite the redundancy of sequences generated by genetic algorithms, this technique is capable of identifying novel artificially generated AMPs with distinct composition and function (Torres and De La Fuente-Nunez, 2019). For instance, evolutionary and genetic algorithms have been used with molecular docking simulations as a fitness function to calculate peptide-receptor interactions followed by AMP optimization through mutations and crossovers (Belda et al., 2005). Thus, a fitness function, which is often ruled by a machine-learning method when sufficient training data are available, provides guidance for AMP design toward regions in sequence space aiming at a higher predicted biological activity (e.g., antibacterial) (Fjell et al., 2012). Within this sequence space, AMP sequences are submitted to modifications to achieve improvements in a “fitness landscape,” which can be explained as a visual evaluation of how promising the modified sequences are, based on the parameter settings (e.g., biochemical activity; structure-activity landscape) (Fjell et al., 2012).

An increasing number of works have used evolutionary and genetic algorithms in combination with NNs, molecular docking, and dynamics as fitness functions for designing AMPs. Fjell et al. (2011), for instance, used a heuristic evolutionary programming method of genetic algorithms to optimize short AMPs (Table 2) (Fjell et al., 2011). In that work, the authors presented an extended version of their previous work (Fjell et al., 2009), in which a software system using ANN and QSAR was developed to predict the activity of 9-amino-acid residue peptides. By using genetic algorithms (Fjell et al., 2011), the authors achieved a 19-fold improvement in AMP identification compared with their previous findings. As a result, ∼0.5% of the peptides generated by genetic algorithms were classified as highly active based on ANN predictions (fitness scores from 0 to 30). The preliminary luminescence assay with *P. aeruginosa PAO1* strain H1001 (containing a luciferase gene cassette *luxCDABE*) allowed the selection of 14 candidate peptides, which were further tested against *P. aeruginosa* PAO1 strain H103, *S. aureus* ATCC25923, MRSA, vancomycin-resistant *Enterococcus faecium*, extended-spectrum β-lactamases (ESBL) *E. coli* and a multidrug-resistant *P. aeruginosa* clinical isolate (Table 2). The peptides were separated into two groups, named GN-1 to -7 and GN-8 to -14. The results demonstrated that some peptides, including GN-2, -4, -5, and -6, showed higher antimicrobial activity against all bacterial strains tested, with MIC values ranging from 2 to 32 μg.mL^–1^ (Fjell et al., 2011). Although this method allowed an improved capacity of identifying novel AMPs, the authors concluded that the *in vitro* activity of the designed AMPs is strongly dependent on the initial peptides’ starting population, despite the achieved fitness score.

More recently, Porto et al. (2018b) reported the use of a genetic algorithm to design AMPs derived from the guava glycine-rich peptide (Pg-AMP1) (Table 2). First, four Pg-AMP1 fragments were used as the initial population and the ratio between hydrophobic moment and α-helix propensity was used as the fitness function (Porto et al., 2018b). A total of 15 peptides, named guavanin 1 to 15, were selected due to their higher fitness values. During screening steps for antimicrobial activity, the variant guavanin 2 was the most potent and, therefore, selected for in-depth analysis. It is worth noting that the determined MICs (initial screening) do not directly correlate with the fitness score for the peptides generated, as also highlighted by Fjell et al. (2011). Guavanin 2 was tested against Gram-positive and -negative bacteria, yeast, and biofilms. The best results were obtained against Gram-negative bacteria, including *E. coli* and *A. baumannii*. In contrast, the same efficacy was not observed against Gram-positive bacteria or yeast. Moreover, among all biofilms tested, only the *C. albicans* biofilms were reduced when treated with guavanin 2. Finally, the *in vivo* activity of guavanin 2 was evaluated against *P. aeruginosa* (skin scarification mouse model – described above). The results showed that guavanin 2 administration (6.25–100 μg.mL^–1^) triggered a 3-log reduction in *P. aeruginosa* counts (Table 2) (Porto et al., 2018b).

The combination of different computational approaches for designing AMPs has also shown promising results. For instance, studies have proposed the design of AMPs by evolutionary multiobjective optimization (Maccari et al., 2013). This method is based on the chemophysical profile of peptides, whose descriptors are coded by QSAR to generate structural and functional statistical models. These models are then used as fitness functions for evolutionary algorithms for designing AMPs. Based on these methods, seven peptide sequences, named GMG_01, GMG_02, GMG_01_SCR, GMG_03, CM18, CM12, GMG_05Z, have been described (Table 2). These peptides (10–18 amino acids) were tested against *S*. *aureus* and *P. aeruginosa*, and the results demonstrated promising antibacterial activities, with MIC values ranging from 0.125 to 16 μM, which is comparable with the most effective AMPs described in the literature. Therefore, the combination of these computational methods conferred high flexibility to AMP design, allowing the generation and selection of highly active drug candidates.

Evolutionary and machine learning algorithms have also been used in combination to design temporin-like AMPs (Table 2). Yoshida et al. (2018) proposed a different design method consisting of three optimization rounds using machine learning and evolutionary algorithms in conjunction with *in vitro* assays (Yoshida et al., 2018). Therefore, the *in vitro* antimicrobial assays were used as fitness functions for designing peptide variants. The natural AMP temporin was used as input sequence and, after three generations of optimization, 256 peptides were tested against *E. coli*, among which 44 peptides presented IC_50_ values (half maximal inhibitory concentration) lower than 4.1 μM (Yoshida et al., 2018). These results revealed that the optimized AMPs are 160-fold more effective than the parent peptide at inhibiting *E. coli* growth. In addition, assays with resistant bacterial strains showed IC_50_ of 1.5–2.0 μM. Differently from the other methods described above, this approach demonstrates how to design potent AMPs without relying on a pre-existing physicochemical database and, therefore, allowing the application of a fitness function based on experimental data (Yoshida et al., 2018).

## Structure Profile in Computer-Made Amps

Antimicrobial peptides feature diverse structural conformations to display antimicrobial activities (Cardoso et al., 2018c). Previous works have reported the clustering of AMPs according to backbone torsion angles, revealing that this class of antimicrobial presents many different folds that could be used to classify them (Fjell et al., 2012). Currently (September 2019), a total of 3128 AMPs from six different kingdoms have been deposited in the APD (Wang et al., 2016). Among the AMPs with structural information, 422 AMPs adopt α-helix conformations, 85 adopt beta structures, 109 present combined helix and beta packed, four present helix and beta unpacked, and 19 show neither helix or beta structures. Moreover, out of the 3128 sequences deposited, only 422 AMPs have tridimensional structures, with 369 structures determined by nuclear magnetic resonance (NMR), and 53 structures by X-ray diffraction (Wang et al., 2016). This means that of every ∼seven sequences deposited, only one has its tridimensional structure determined by experimental techniques.

As described above, the majority of AMPs deposited in public databases adopt α-helix structures, usually in membrane-like environments. Helicity has commonly been associated with the effectiveness of many AMPs reported to date, as it has been shown, in some cases, to improve AMP specificity (Huang et al., 2014; Khara et al., 2015). Therefore, diverse computational approaches for designing AMPs consider the helical content as a crucial determinant for generating improved AMPs. Based upon the data summarized here, QSAR-designed AMPs, including dadapin peptides (Rončević et al., 2019), undergo a coil-to-helix transition (Table 2) from hydrophilic to hydrophobic or membrane-like conditions [e.g., 2,2,2-trifluoroethanol (TFE), sodium dodecyl sulfate (SDS), and dodecylphosphocholine (DPC) micelles, as well as liposomes]. Moreover, the *de novo* design of peptides with greater helicity has resulted in broad-spectrum antibacterial activities compared with AMPs with low helical content (Table 2) (Faccone et al., 2014). Furthermore, structure-guided *de novo* design for short, pore-forming AMPs has shown that these peptides required α-helix arrangements to penetrate bacterial membranes successfully, leading to membrane disruption and, finally, cell death (Chen et al., 2019). Similar findings were reported for a guava derived AMP, named guavanin 2, designed based on a genetic algorithm (Table 2) (Porto et al., 2018b). This peptide was shown to adopt α-helix in membrane conditions, which was further correlated with the ability of this peptide in causing membrane disruption and triggering hyperpolarization (Porto et al., 2018b).

Indeed, the organization of AMPs in helical structures has resulted, in most cases, in biologically active molecules toward bacteria. However, we cannot discard the increasing numbers of reports that highlight AMP flexibility as a promising scaffold for multifunctional properties in the context of bacterial and biofilm infections (Pukala et al., 2004; Cardoso et al., 2018a). For instance, AMPs designed by automated antimicrobial pattern insertion, including the above-mentioned EcDBS1R5 and mastoparan-R1/R4, have shown that flexible (Table 2), helical structures may trigger enhanced antibacterial, antibiofilm, and anti-infective properties (Cardoso et al., 2018a; Oshiro et al., 2019). EcDBS1R5 secondary structure was investigated in different mimetic conditions and its tridimensional structure determined in 30% TFE. As a result, a short central α-helical segment with flexible termini was reported and correlated with the antibacterial properties observed for this peptide (Cardoso et al., 2018a). In recent work with mastoparan peptides, mastoparan-L was used as a template sequence for the generation of mastoparan-R1 and R4 (Oshiro et al., 2019). When evaluated structurally, NMR and temperature coefficient experiments revealed that the levels of structural stability of the peptides follow the order: mastoparan-L > R4 > R1. Interestingly, the most flexible peptide, mastoparan-R1, presented not only antibacterial and antibiofilm activities but was also the most active peptide *in vivo* (Oshiro et al., 2019).

Apart from α-helical AMPs (regardless of their levels of flexibility), some computationally designed peptides present short sequences with specific amino acid repetitions, including tryptophan and arginine, which do not favor α-helix formation. The immunomodulatory and antibiofilm peptide IDR-1018 (VRLIVAVRIWRR-NH_2_), for instance, has been used as a starting sequence for QSAR methods aiming at generating peptide candidates for antibiofilm therapies. The secondary structure of IDR-1018 has been investigated in different conditions, revealing high structural plasticity (Wieczorek et al., 2010). Moreover, NMR studies were carried out and a central turn of α-helix in the presence of DPC was reported for IDR-1018. MD simulations, in which IDR-1018 structure varied from short α-helix to random coil and beta conformations, further confirmed the structural plasticity of this peptide. Therefore, considering the sequence similarity between IDR-1018 and AMPs generated by QSAR models (Haney et al., 2018) and genetic algorithms (Fjell et al., 2011), it may be expected that these peptides should present similar structural behavior.

The findings summarized in this section indicate that, although helical structures have long been used as an important feature for designing AMPs, computer-aided methods can generate AMP candidates with different structural profiles, thus providing novel structural scaffolds that may lead to different biological activities in the future.

## Conclusion – Are We Generating Effective Drug Candidates?

As described in the previous topics, diverse computational tools have been developed and applied alone or in combination to design novel peptide-based drug candidates. So far, these methods have contributed to an increasing number of AMP sequences deposited in databases, thus providing useful information for future AMP design studies. Moreover, when allied with high throughput screening methods for antibacterial and hemolytic activities, including colorimetric assays (Kolusheva et al., 2000) and SPOT-synthesis of peptide arrays on cellulose membranes (Figure 1) (Hilpert et al., 2007), the chances of selecting promising AMPs are higher, which has also been confirmed by *in vivo* assays using animal models of infection.

The SPOT synthesis of peptide arrays, for instance, has been successfully used as a methodology for a rapid investigation of single amino acid substitution libraries at every position in a target peptide. From this point, studies have reported high throughput screening for antibacterial, antibiofilm, hemolytic, and immunomodulatory properties (Haney et al., 2015). All of this information can be further used for substitution matrices to guide the development of a new generation of optimized peptide-based drugs. In addition, SPOT-synthetized peptides have also been evaluated in luminescence assays, in which an engineered luminescent bacterial strain (e.g., *P. aeruginosa* H1001) is submitted to different concentrations of the peptide candidates, followed by luminescence measurement (Hilpert et al., 2009). Interestingly, apart from the high throughput screening for biological properties, AMP candidate sequences have also been screened for their ability to recognize bacterial membranes and based on their mechanism of action (Xie et al., 2006; Von Gundlach et al., 2016). For instance, Xie et al. (2006) developed a ribosome display system to establish peptide/ribosome/mRNA complexes that were further evaluated on immobilized model membranes, aiming at selecting specific sequences that recognize bacterial membranes. Finally, studies have shown the usefulness of small-angle X-ray scattering (SAXS) as a high throughput method to classify AMPs’ mechanisms of action. It has been reported that SAXS provides fast and reliable information on the ultrastructural changes that a particular antimicrobial agent (e.g., AMPs) causes on pathogenic bacteria (Von Gundlach et al., 2016). Therefore, SAXS can be used not only to classify AMP modes of action but also to compare them with those from conventional antibiotics, which in turn may facilitate the development of multi-target AMP candidates.

Although computer-aided design and high throughput screening of AMPs have significantly evolved over the years, a critical question remains: are we generating effective drug candidates? This can be an intriguing and paradoxical question. If we think about the enrichment of peptide sequence databases that could be used as scaffolds/templates for future peptide optimization, we have indeed greatly contributed to the field of peptide-based antibiotics. In contrast, however, many studies focus only on AMP generation by means of computers followed by their in-depth characterization, but without a subsequent effort to translate these candidates to clinical trials. As a consequence, the current scenario reveals the discrepancy between the numbers of AMP sequences identified/generated and fully characterized for function/structure and the real outcomes in the clinical trials, which also includes computationally designed AMPs.

Among the challenges involved in developing AMPs for clinical applications we can mention: (i) the divergence between *in vitro* and *in vivo* antibacterial assays in terms of biological complexity, thus compromising accurate prediction of anti-infectious potential in AMPs at clinical level (Bjorn et al., 2012; Maiti et al., 2014); (ii) AMP susceptibility to enzymatic degradation, thus compromising the bioavailability of these antimicrobials, which represents an obstacle for oral/intravenous administration (Vlieghe et al., 2010; Cardoso et al., 2018b); and (iii) cost of synthesis compared with other small molecule drugs (Bray, 2003).

Even considering these obstacles, a few AMPs (non-computationally designed) have reached advanced trials and have been introduced in the market. Among them, polymyxin antibiotics are the most well-characterized AMPs for clinical use (Landman et al., 2008). In addition, pexiganan (an analog from the frog-derived magainin) and iseganan (derived from protegrin 1) are in phase III trials for infected diabetic foot ulcers and oral mucositis, respectively [please check the clinical trial identifiers (CTIs): NCT00563394 and NCT00563433 for pexiganan; and NCT00022373 for iseganan]. Moreover, an AMP derived from bovine indolicidin has achieved phase II/III trial for catheter infections and rosacea (CTI: NCT00231153 and NCT01784133). In phase II trials, PXL01 (derived from human lactoferricin) has been used for the prevention of post-surgical adhesion formation in hand surgery (CTI: NCT01022242); and PAC-113 (derived from the human saliva histatin 3) has been used to treat oral candidiasis in HIV seropositive patients (CTI: NCT00659971). Finally, phase I/II trials include lytixar for uncomplicated Gram-positive skin infections, impetigo, and nasal colonization with *S. aureus* (CTI: NCT01223222, NCT01803035, and NCT01158235); and hLF1-11 for bacteremia and fungal infections in immunocompromised hematopoietic stem cell transplant recipients (CTI: NCT00509938). For a more extensive review of these peptide-based drugs, see Mahlapuu et al. (2016).

In summary, although the data here summarized for computationally designed AMPs present a significant advance in terms of sequence optimization, structure diversity, *in vivo* activity, and improved therapeutic indexes, these peptides have not yet achieved more advanced trials. On the other hand, it is worth noting the huge advance in peptide development using computer methods and how this strategy has enriched public databases with crucial information for future peptide-based drug design. Indeed, all the information provided by these methods, including QSAR methods, *de novo* design, linguistic models, pattern insertion, and evolutionary algorithm, is of enormous value for future studies using these AMPs as model scaffolds to achieve higher effectiveness toward bacteria *in vivo*, improved bioavailability, and cell specificity. Moreover, considering the rapid development of computational tools over the years, it is expected that highly accurate methods will help researchers to improve scoring functions for designing and predicting AMP sequences at low cost. Taken together, all these features will certainly assist an increasing number of computationally designed AMPs to evolve from database sequences to real, effective drug candidates that are more likely to reach the market in upcoming years.

## Author Contributions

MC, RO, SR, GR, KO, and EC wrote the manuscript. MC and KO idealized and organized the figure. MC and OF corrected the manuscript.

## Conflict of Interest

The authors declare that the research was conducted in the absence of any commercial or financial relationships that could be construed as a potential conflict of interest.
